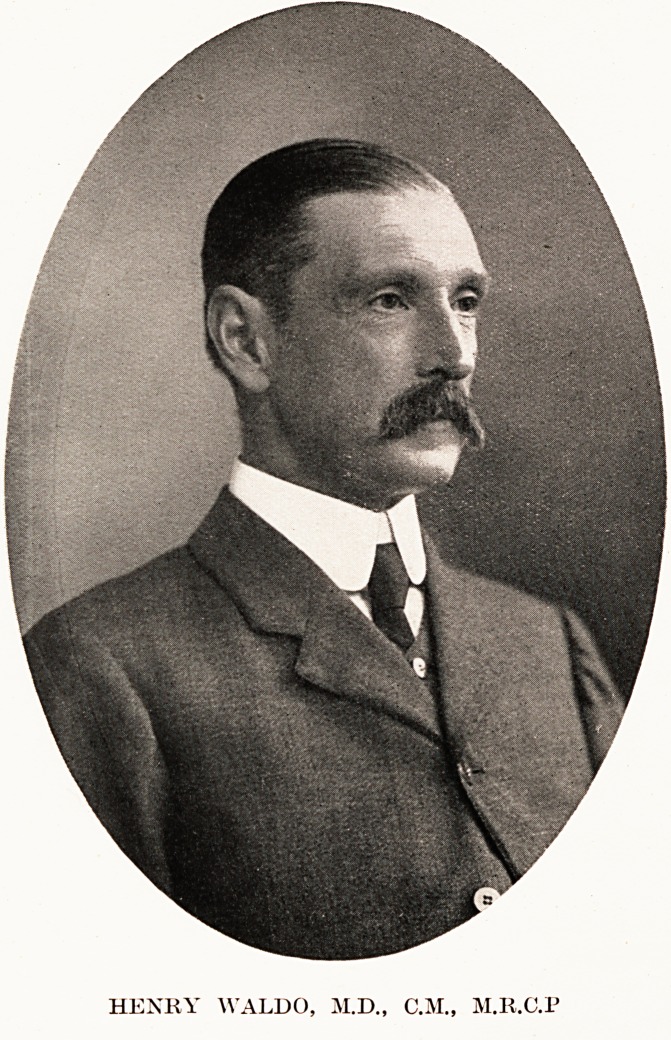# Henry Waldo

**Published:** 1935

**Authors:** P. Watson-Williams


### HENRY WALDO, M.D., C.M., M.R.C.P.

Dr. Henry Waldo, who was born in Bristol in 1846, died on January 25th, 1935, at the close of a long and honourable career in the service of his native city. On leaving Bristol Grammar School in 1867 he entered the Bristol Medical School, and though this was sixty-eight years ago, we are glad to say that two of his fellow-students who entered a year earlier are still with us—Dr. J. E. Shaw, Consulting Physician to the Bristol Royal Infirmary, and Dr. Conrad Fitz-Gerald of Newfoundland.

Dr. Waldo continued his medical studies at St. Bartholomew's Hospital and at the University of Aberdeen, graduating M.B., C.M., in 1871, and proceeding to M.D. two years later; he also took the diploma of M.R.C.S. and in 1888 the M.R.C.P. After having held the post of Resident Medical Officer at the Royal General Dispensary in London he returned to his native city, and in 1873 was appointed Assistant Physician to the Bristol Royal Infirmary and within three years became full Physician. Dr. Waldo succeeded Dr. Fairbrother, who had resigned in accordance with the twenty years rule on June 27th, 1876. When Dr. E. Long Fox resigned on August 14th, 1877, the rules for staff appointments were altered, the tenure of office by physicians and surgeons being made subject to an age limit. This replaced the old rule of 1843, under which they were elected on condition that they did not hold office for more than twenty years.

Dr. Waldo being one of the first physicians appointed under the new rules, continued to serve for thirty years. He was appointed Consulting Physician in 1906, holding this office for twenty-nine years. Thus he was on the staff of the Royal Infirmary for sixty-one years in all.

In 1902 Dr. Waldo was President of the old Bath and Bristol Branch of the British Medical Association, and in 1907 he was President of the Bristol Medico-Chirurgical Society.

HENRY WALDO, M.D., C.M., M.R.C.P

In the latter year he was President of the Dermatological Society of Great Britain, a recognition of his work in charge of the new Department for Diseases of the Skin at the Royal Infirmary, to which he had been appointed in 1880.

The writer was for many years closely associated with Dr. Waldo as a colleague on the medical staff, and learnt to appreciate his sound training, sterling qualities and lovable disposition, and as so well expressed by his old friend and fellow-student, Dr. L. A. Weatherly, " he was quite the type of a family doctor, loved and respected by all his patients and their confidential friends."

His younger brother, the late Dr. F. J. Waldo, was Coroner for the City of London and Borough of Southwark.

Dr. Waldo became a widower twenty-six years ago, but he is survived by his two sons (one of whom, Dr. H. C. Waldo, is Medical Superintendent of the Nottinghamshire Mental Hospital) and three daughters.

P. WATSON-WILLIAMS.

## Figures and Tables

**Figure f1:**